# PSegNet: Simultaneous Semantic and Instance Segmentation for Point Clouds of Plants

**DOI:** 10.34133/2022/9787643

**Published:** 2022-05-23

**Authors:** Dawei Li, Jinsheng Li, Shiyu Xiang, Anqi Pan

**Affiliations:** ^1^State Key Laboratory for Modification of Chemical Fibers and Polymer Materials, College of Information Sciences and Technology, Donghua University, Shanghai 201620, China; ^2^Engineering Research Center of Digitized Textile & Fashion Technology, Ministry of Education, Donghua University, Shanghai 201620, China; ^3^College of Information Sciences and Technology, Donghua University, Shanghai 201620, China

## Abstract

Phenotyping of plant growth improves the understanding of complex genetic traits and eventually expedites the development of modern breeding and intelligent agriculture. In phenotyping, segmentation of 3D point clouds of plant organs such as leaves and stems contributes to automatic growth monitoring and reflects the extent of stress received by the plant. In this work, we first proposed the Voxelized Farthest Point Sampling (VFPS), a novel point cloud downsampling strategy, to prepare our plant dataset for training of deep neural networks. Then, a deep learning network—PSegNet, was specially designed for segmenting point clouds of several species of plants. The effectiveness of PSegNet originates from three new modules including the Double-Neighborhood Feature Extraction Block (DNFEB), the Double-Granularity Feature Fusion Module (DGFFM), and the Attention Module (AM). After training on the plant dataset prepared with VFPS, the network can simultaneously realize the semantic segmentation and the leaf instance segmentation for three plant species. Comparing to several mainstream networks such as PointNet++, ASIS, SGPN, and PlantNet, the PSegNet obtained the best segmentation results quantitatively and qualitatively. In semantic segmentation, PSegNet achieved 95.23%, 93.85%, 94.52%, and 89.90% for the mean Prec, Rec, F1, and IoU, respectively. In instance segmentation, PSegNet achieved 88.13%, 79.28%, 83.35%, and 89.54% for the mPrec, mRec, mCov, and mWCov, respectively.

## 1. Introduction

Plant phenotyping is an emerging science that connects genetics with plant physiology, ecology, and agriculture [1]. It studies a set of indicators formed by the dynamic interaction between genes and the growth environment to intuitively reflect the growth of plants [2]. The main purpose of the research is to accurately analyze the relationship between phenotypes and genotypes by means of computerized digitization, to improve the understanding of complex genetic traits, and eventually expedite the development of modern breeding and precision agriculture [3–5]. Generally speaking, the analysis of plant phenotypes mainly focuses on organs, including the aspects such as leaf characteristics, stem characteristics, fruit traits, and root morphology. As the organ with the largest surface area, leaves serve for the main place of photosynthesis and respiration [6]. Therefore, the leaf area, leaf length, width, and the leaf inclination are among the most critical phenotypic factors [7]. In addition to leaves, the stem system not only forms the skeleton of the plant structure but also spatially connects all other organs such as leaves, flowers, and fruits. The phenotyping of the stems can reflect the extent of stress received by the plant.

The key to plant phenotyping is to segment plant organs efficiently and correctly. Since the 1990s, a flow of researches have emerged upon the task of plant organ segmentation, especially the leaf segmentation for disease recognition. The 2D image-based phenotyping is usually based on traditional image processing, machine learning, and pattern recognition algorithms, such as the threshold-based segmentation [8, 9], edge detection [10, 11], region growing [12, 13], clustering [14, 15], and their combinations and extensions [16–20]. In recent years, deep learning based on convolutional neural networks (CNNs) has reached state of the art on image classification and image segmentation [21–23]. References [24–29] applied image deep learning networks to segment fruits and leaves from plant images. However, the 2D phenotyping methods usually deal with simple rosette plants (e.g., *Arabidopsis* or tobacco) or several monocotyledonous plants with fewer leaves (e.g., wheat and maize). The main reason is that a 2D image is taken from only one viewing angle, missing the depth information. And the occlusion and overlapping between leaves in the canopy bring huge challenges to the segmentation algorithms based on 2D images. Moreover, images cannot fully describe the complete spatial distribution of the plant structure, resulting in a less reliable statistical significance of the measured phenotypic traits.

Compared with images, 3D models not only contain the information of color and texture but can also carry the most important information—the depth. Depth directly overcomes the problems caused by occlusion and overlapping, which are becoming the basis for high-precision phenotypic measurement. In recent years, with the development of low-cost and high-precision 3D imaging technology, plant phenotyping methods based on depth images or point clouds are quickly emerging. As the 3D imaging technique with the highest precision, Light Detection and Ranging (Lidar) is now widely used for 3D reconstruction and phenotyping of tall trees [30, 31], maize [32, 33], cotton [34], and several other cash crops [35–37]. 3D sensors based on Structured Light and Time-of-Flight (ToF) have also become two important means for 3D phenotyping on plants due to their remarkable real-time performances [38, 39]. References [40, 41] reconstructed and analyzed a variety of greenhouse crops and cash crops using binocular stereo vision. References [42, 43] carried out 3D reconstruction and phenotypic analysis on crops by using the multiview stereo (MVS) technique. Miao et al. designed a toolkit—Label3DMaize [44], for annotating 3D point cloud data of maize shoots; the toolkit facilitates the preparation of manually labeled maize 3D data for training and testing on machine learning models.

Despite the precise 3D data, how to effectively separate plant individuals from the cultivating block and how to further divide each plant into organs to calculate phenotypic parameters are two difficult tasks in phenotyping. Unsupervised leaf segmentation on 3D point clouds has already begun to attract interests. Paproki et al. [45] improved the point cloud mesh segmentation algorithm and proposed a hybrid segmentation model that could adapt to the morphological differences among different individuals of cotton, and they achieved the separation of leaves from stems. Duan et al. [46] used the octree algorithm to divide the plant point cloud into small parts and then manually merged each part into a single organ according to their spatial topological relationships. Itakura and Hosoi [47] utilized the projection method and the attribute extension for leaf segmentation; they also tested the segmentation accuracy of seedlings of 6 types of plants. Su et al. [48] and Li *et al.* [49] used the Difference of Normal (DoN) [50] operator to segment leaves in point clouds of magnolia and maize, respectively. In addition, Zermas et al. [51] proposed to use node information in the 3D skeleton of plant to segment the overlapping maize leaves. The abovementioned point cloud segmentation and phenotyping techniques still lack generality on segmentation of different crop species with diverse leaf shapes and canopy structures. Meanwhile, their applications are sometimes restricted by the complicated parameter tuning in segmentation. Pan et al. [52] and Chebrolu *et al*. [53] used spatiotemporal matching to associate the organs in growth for phenotypic growth tracking.

Designing a general 3D segmentation method for multiple plant species at different growth stages is the current frontier of 3D plant phenotyping. With the recent breakthrough in artificial intelligence, deep learning-based segmentation methods for unorganized and uneven point clouds are becoming popular across both academics and the agricultural industry. Previous studies mainly focused on the multiview CNNs [54–58] that understand the 3D data by strengthening the connection between 2D and 3D by CNNs on images. However, two issues exist in multiview CNNs, i.e., it is hard to determine the angle and quantity of projection from a point cloud to a 2D image, and the reprojection from the segmented 2D shapes back to the 3D space is not easy. Some studies resorted to a generalization from 2D CNNs to the voxel-based 3D CNNs [59–63]. In 3D CNN, the point cloud is first divided into a large number of voxels and 3D convolutions are used to achieve direct segmentation on the point cloud. However, the computational expense of this method is high. PointNet [64] and PointNet++ [65] operate directly on points and are able to simultaneously conduct classification and semantic segmentation at point-level. Since then, improvements on the PointNet-like framework were made to enhance the network performance mainly by optimizing and/or redesigning the feature extraction modules [66]. Masuda [67] applied PointNet++ to the semantic segmentation of tomato plants in greenhouse and further estimated the leaf area index. Li et al. [68] designed a PointNet-like network to conduct semantic and instance segmentation on maize 3D data. Similarity group proposal network (SGPN) [69] devised a similarity matrix and group proposals to realize simultaneous instance segmentation and semantic segmentation of point clouds. Graph neural networks (GNNs) [70–74] obtained information between adjacent nodes by converting the point cloud into a connective graph or a polygon mesh.

So far, deep learning has becoming a promising solution for high-precision organ segmentation and phenotypic trait analysis of plant point clouds. However, several problems are yet to be solved—(i) the lack of a standardized downsampling strategy for point clouds that are specially prepared for deep learning; (ii) the network design for multifunctional point cloud segmentation is challenging—e.g., a network is hard to keep balance between the organ semantic segmentation task and the instance segmentation task; and (iii) the lack of generalization ability among different species; e.g., a good segmentation network for monocotyledonous plants may not work properly on dicotyledonous plants.

To address the above challenges, a deep learning network—PSegNet, was designed to simultaneously conduct plant organ semantic segmentation and leaf instance segmentation on a manually labeled point cloud dataset of multiple species. PSegNet obtained state-of-the-art results on two kinds of dicotyledonous plants (tobacco and tomato) and a monocotyledonous plant—sorghum. The detailed contributions are stated as follows:

- We proposed Voxelized Farthest Point Sampling (VFPS), a novel point cloud downsampling strategy that possesses advantages from both Voxelization-based Sampling (VBS) and Farthest Point Sampling (FPS). VFPS is suitable to be used to prepare diversified dataset for training of deep neural networks because it can easily augment point cloud data by the random initialization in sampling. Ablation experiments “A6” showed that the proposed VFPS strategy significantly improved the accuracies of organ semantic segmentation and instance segmentation for several varieties of crops by contrasting with the traditional FPS
- A deep learning network—PSegNet, was specially designed for segmenting point clouds of several species of plants. After training on the dataset prepared with VFPS, the network can simultaneously realize the semantic segmentation of the stem class and the leaf class and the instance segmentation for each single leaf. Comparing to several mainstream deep learning networks such as PointNet++ [65], ASIS [75], SGPN [69], and PlantNet [76], our PSegNet obtained the best segmentation results qualitatively and quantitatively. The effectiveness of the modules in the architecture of PSegNet, including the Double-Neighborhood Feature Extraction Block (DNFEB), the Double-Granularity Feature Fusion Module (DGFFM), and the Attention Module (AM), was verified separately by the ablation study

The notations and nomenclatures used in this paper are summarized in Table 1. The rest of the paper is arranged as follows. Materials and related methods are explained in Section 2. Comparative experiments and results are given in Section 3. Some further discussions and analysis are provided in Section 4. The conclusion is drawn in the last section.

## 2. Materials and Methods

### 2.1. Point Cloud Sampling

There are usually tens of thousands of points in a high-precision point cloud, and the processing of such complicated point clouds will inevitably incur a huge computation burden. Moreover, the majority of modern neural networks for 3D learning only accept standardized input point clouds, i.e., a fixed number of points for all point clouds. Therefore, it is necessary to carry out sampling before substantial processing and modeling. There are two commonly used methods for point cloud downsampling—the Farthest Point Sampling (FPS) [65] and the Voxel-based Sampling (VBS) [77]. FPS first takes a point from the original point cloud *P* to form a point set *A*, and each time then traverses all points of the set *P*\*A* to find the farthest point *p* from *A*. Finally, the point *p* is taken out from *P*\*A* into *A*, and the iteration ends till the point set *A* has reached the number limit. This method can maintain the local density of the point cloud after sampling with a moderate computational cost. The disadvantage of FPS is that it may easily lose details of areas that are already sparse and small. VBS constructs voxels in the 3D space of the point cloud, and the length, width, and height of the voxel are defined as three voxel parameters *l*_*x*_, *l*_*y*_, *l*_*z*_, respectively. VBS uses the center of gravity of each voxel to replace all original points in that voxel to achieve downsampling. Despite the high processing speed, there are two main disadvantages of VBS—(i) the three parameters for the scale of voxelization need to be adjusted according to the density and the size of the point cloud, making the number of points after VBS sampling to be uncertain. Therefore, VBS cannot be directly adopted on the batch processing of a large point cloud dataset. And (ii) VBS creates evenly distributed point clouds, which is unfavorable for the training of deep neural networks whose performances rely on the diversity of the data distribution.

**Figure 1:**
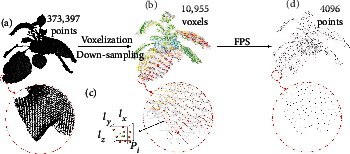
Schematic diagram of the VFPS strategy. The leftmost point cloud (a) is an original tobacco point cloud that contains a total of 373,397 points. First, we set a number object for downsampled point cloud, e.g., *N* = 4096. Then, the VBS with parameters *l*_*x*_ = *l*_*y*_ = *l*_*z*_ = 3cm is applied to the original point cloud to form a point cloud (b) containing 10955 voxels. Each voxel is represented by the center of gravity of points in the voxel, and a voxel example is enlarged in (c). At last, FPS is applied on this temporary voxelized point cloud to generate the final result (d) with an exact 4096 points.

After studying the strengths and weaknesses of FPS and VBS, a new point cloud downsampling strategy—Voxelized Farthest Point Sampling (VFPS), is proposed. The VFPS strategy can be divided into three steps shown as Figure 1. The first step is to determine *N*, the number of downsampled points. The second step is to adjust the voxel parameters to conduct VBS on the original point cloud to generate a point cloud having slightly more points than *N*. The last step is to apply FPS on this temporary voxelized point cloud, and downsample is a result with *N* points. In all experiments of this paper, we fix *N* at 4096. VFPS possesses advantages from both VBS and FPS; it can not only generate standardized points but can also easily generate diversified samples from the same original point cloud by starting FPS from a randomly chosen point, which is highly suitable to be used as a data augmentation measure for the training of deep learning networks.

### 2.2. Network Architecture

**Figure 2:**
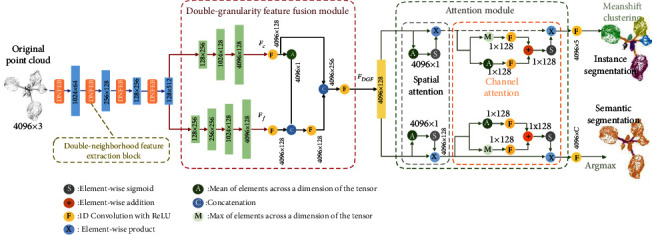
The architecture of PSegNet. The network is mainly composed of three parts. The front part has a typical encoder-like structure in deep learning. Four consecutive Double-Neighborhood Feature Extraction Blocks are applied in the front part computation, and the feature space is downsampled before each DNFEB to condense the features, respectively. The middle part is the Double-Granularity Feature Fusion Module, which fuses the outputs of two decoders with different feature granularity to obtain the mixed feature *F*_DGF_. In the third part of PSegNet, the features flow into two directions that, respectively, correspond to two tasks—instance segmentation and semantic segmentation. Spatial attention and channel attention mechanisms are sequentially applied on each feature flow.

The overall architecture of PSegNet is shown in Figure 2. The end-to-end network is mainly composed of three parts. The front part has a typical encoder-like structure that is frequently observed in deep neural networks, and the front part contains four consecutive Double-Neighborhood Feature Extraction Blocks (DNFEBs) for feature calculation. Before each DNFEB, the feature space is downsampled to reduce the number of points and to condense the features. The design of the middle part of the network is inspired from Deep FusionNet [78]. We name this part as Double-Granularity Feature Fusion Module (DGFFM), which first executes two parallel decoders, in which the upper decoder is a coarse-grained decoder, while the lower decoder has more layers, representing a fine-grained process. At the end of DGFFM, concatenation operation and convolutions are carried out to realize feature fusion. The third part of the network has a double-flow structure. The feature flow of the upper branch and the feature flow of the lower branch correspond to the instance segmentation task and semantic segmentation task, respectively. Features on each flow of the third part pass through the Attention Module (AM) that contains a spatial attention and a channel attention operation, so that the two flows can aggregate desirable semantic information and instance information, respectively. In the output part of PSegNet, the semantic flow obtains the predicted semantic label of each point through the argmax operation on the last feature layer to complete the semantic segmentation task. The instance flow performs MeanShift clustering [79] on the last feature layer to realize the instance segmentation.

### 2.3. Double-Neighborhood Feature Extraction Block

**Figure 3:**
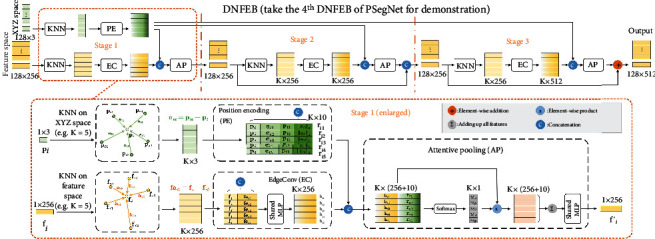
Demonstration of DNFEB. The feature dimensions in DNFEB vary with their positions in the PSegNet, and in this figure, we only display feature dimensions of the 4^th^ DNFEB. A standard DNFEB contains three similar stages. The calculation process of stage 1 is enlarged in the lower part of the figure. On stage 1, for any point *i* in the feature space, we find its K-nearest neighbors in the initial *XYZ* space and in the current feature space, respectively. Secondly, position encoding is carried out for K-nearest neighbors in *XYZ* space to form a low-level feature encoding of the local region. At the same time, EdgeConv is carried out for the K-nearest neighbors in the current feature space to form a high-level feature representation of the local region. Finally, after concatenating the low-level and high-level local features, the new feature vector of the current point *i* is output after the calculation of the Attentive Pooling operation.

DNFEB was designed to improve the feature extraction in the front part of PSegNet. The front part contains 4 consecutive DNFEBs. And in this encoder-like front part, DNFEB follows immediately after a downsampling of the feature space each time. DNFEB has two characteristics: (i) it pays attention to the extraction of both high-level and low-level features at the same time; (ii) by continuously aggregating the neighborhood information in the feature space, the encoder can realize comprehensive information extraction from the local scale to the global one. The detailed workflow of DNFEB is shown in Figure 3, which contains two types of K-nearest neighborhood calculations and three similar stages in a row.

The lower part of Figure 3 enlarges the detailed calculation steps of the stage 1 of DNFEB. Take the *i*-th point as example, its original *XYZ* space coordinate (the low-level feature space) is represented by **p**_*i*_, and the feature vector of its current feature space is **f**_*i*_. The K-nearest neighbors searched in the initial *XYZ* space are defined as a set {**p**_*i*1_, ⋯, **p**_*iK*_}, while the K-nearest neighbors in the current feature space are denoted by {**f**_*i*1_, ⋯, **f**_*iK*_}. The low-level neighborhood features are calculated by the Position Encoding (PE) operation to obtain the encoded low-level neighborhood feature set {**r**_*i*1_, ⋯, **r**_*iK*_}, in which the feature vector is calculated by (1):

(1) $$\begin{array}{c} r_{iK}=p_{i}⋃p_{ik}⋃\left(p_{i}-p_{ik}\right)⋃{\left\|p_{i}-p_{ik}\right\|}_{2}. \end{array}$$

In Equation (1), the operator ∪ represents vector concatenation, and ‖·‖_2_ represents the L2-norm. Therefore, **r**_*iK*_ is a 10-dimensional low-level vector, representing the position information of the *k*-th point near **p**_*i*_. The neighborhood of the current feature space is calculated by the EdgeConv (EC) [74] operation to obtain a high-level neighborhood feature set {**h**_*i*1_, ⋯, **h**_*ik*_}. The calculation method of **h**_*ik*_ in the set is given in (2).

(2) $$\begin{array}{c} h_{ik}=\text{MLP}\left[f_{i}⋃\left(f_{i}-f_{ik}\right)\right], \end{array}$$

where MLP[·] represents a multilayer perception operation with shared parameters.

The encoded low-level neighborhood {**r**_*i*1_, ⋯, **r**_*ik*_} is concatenated with the high-level neighborhood {**h**_*i*1_, ⋯, **h**_*ik*_}, and the combination then aggregates into a single feature vector **f**_*i*_′ by the Attentive Pooling (AP) operation. The calculation process can be represented by the following equation.

(3) $$\begin{array}{c} f_{i}^{\prime }=\text{MLP}\left[\overset{K}{\underset{k=1}{\sum }}w_{ik}\cdot \left(h_{ik}⋃r_{ik}\right)\right], \end{array}$$

in which the weight set {*w*_*ik*_|*k* = 1, ⋯*K*} is obtained by Softmaxing on the concatenated features of the low-level and high-level feature sets. The following addition of all features in the neighborhood realizes information aggregation. Equation (3) can be regarded as a generalized form of average pooling. Due to the introduction of attention mechanism, its effect is better than ordinary average pooling, which has become a standard operation in deep learning. After the calculation of the AP module, the vector **f**_*i*_′ becomes the output of the *i*^th^ point on stage 1. After all points have completed the AP process on stage 1, the set {**f**_1_′, ⋯, **f**_*N*_′} will become the input of stage 2 of DNFEB to continue the calculation. Though the three stages seem similar, it should be noted that several tiny differences exist among them. For example, the output of stage 1 is concatenated with the output of stage 2; however, the output of stage 2 is directly added with stage 3 in order to reduce the amount of parameters. And, there is also no skip connection on stage 1.

### 2.4. Double-Granularity Feature Fusion Module

Deep FusionNet [78] believes that both the coarse-grained voxel-level features and the fine-grained point-level features are of great significance to feature learning. This network strengthens its feature extraction ability after fusing the features under two different granularities. Inspired by Deep FusionNet, we design a Double-Granularity Feature Fusion Module (DGFFM) in the middle part of our PSegNet. In DGFFM (shown in Figure 2), two parallel decoders are created to construct feature layers with two different learning granularities, in which the upper decoder with less layers simulates the coarse-grained learning process, while the lower decoder with more layers simulates the fine-grained learning. After obtaining the coarse-grained feature map *F*_*c*_ and the fine-grained feature map *F*_*f*_, the module combines them through operations such as average pooling and feature concatenation and finally obtains the aggregated feature *F*_DGF_ after 1D convolution with ReLU. The output of DGFFM can help to improve the performances of the semantic and instance segmentation tasks that follow.

### 2.5. Attention Module and Output Processing

Attention has become a promising research direction in deep learning field, and although it has been widely applied to 2D image processing [80–84], the use of attention mechanism on 3D point cloud learning networks is still in its infancy. Inspired by [81], we design an Attention Module (AM) that contains two subattention mechanisms, i.e., the spatial attention (SA) and the channel attention (CA). Spatial attention (SA) tends to highlight points that can better express meaningful features among all input points and gives higher weights to those more important feature vectors for strengthening the role of key points in the point cloud representation. Channel attention (CA) tends to focus on several dimensions (channels) of the feature vector that encode more interesting information. The importance of each feature channel is automatically judged via pooling along the feature channel direction, and then, each channel is given a different weight according to its importance to strengthen the important feature dimensions and meanwhile suppress the unimportant dimensions.

In the structure of AM, the feature calculation is divided into two parallel ways, the feature flow of the upper branch performs the instance segmentation task, while the feature flow of the lower branch corresponds to the semantic segmentation task. Both feature flows first pass the calculation of SA and then the CA. Although the calculation measures are the same for the two flows, the parameters of parallel convolutions are not shared. In SA, we create a vector of 4096 × 1 by carrying out average pooling on the output feature 4096 × 128 from DGFFM and then conduct sigmoid function on the vector to obtain the spatial weight vector. By multiplying the weight vector with the DGFFM feature, the feature strengthened by SA is obtained, and its size is 4096 × 128. In the following CA, we obtain two vectors with size 1 × 128 by averaging pooling and maxing pooling along the channel direction of the SA output, respectively. And then, two vectors are added together after 1D convolutions. Finally, we use sigmoid function to obtain a 1 × 128 channel weight vector. By multiplying the weight vector with the pooled feature vector, we obtain the strengthened feature vector of the SA mechanism, which is also the output of a flow of the AM module.

For the point cloud instance segmentation task, the instance flow outputs a 4096 × 5 feature map after AM calculation with an extra 1D convolution. Then, the MeanShift clustering algorithm [79] is used on the feature map to generate the instance segmentation result. During training, the loss of instance segmentation is calculated immediately after clustering. For the semantic segmentation task, by using an extra 1D convolution and an Argmax operation on the semantic flow output feature of AM, we obtain a result of 4096 × *C* one-hot encoded feature, in which *C* represents the number of semantic classes. The loss of semantic segmentation is calculated here.

### 2.6. Loss Functions

The loss functions play an indispensable role in the training of deep learning networks, and our PSegNet uses carefully designed different loss functions to supervise different tasks at the same time. In the semantic segmentation task, we use the standard cross-entropy loss function *L*_sem_, which is defined as follows:

(4) $$\begin{array}{c} L_{\text{sem}}=-\overset{N}{\underset{i=1}{\sum }}\overset{C}{\underset{j=1}{\sum }}x_{j}^{\prime }\left(i\right)\log x_{j}\left(i\right), \end{array}$$

in which *x*_*j*_(*i*) is the predicted probability that the current point **p**_*i*_ belongs to class *j*, and *x*_*j*_′(*i*) is the one-hot encoding of the true semantic class of the point. If the point truly belongs to the category  *j*, the value of *x*_*j*_′(*i*) is 1, otherwise 0. In the instance segmentation task, the number of instances in an input point cloud is variable. Therefore, we use a comprehensive loss function that includes three weighted sublosses under an uncertain number of instances to supervise the training. The equation for the instance loss *L*_ins_ is given as follows:

(5) $$\begin{array}{c} L_{\text{ins}}=\alpha \cdot L_{s}+\beta \cdot L_{d}+\gamma \cdot L_{\text{reg}}. \end{array}$$

The weights of sublosses *L*_*s*_, *L*_*d*_, *L*_reg_ in the total loss are represented by *α*, *β*, *γ*, respectively. *L*_*s*_ is devised to make it easier for the points of the same instance label to gather together. The function *L*_*d*_ is to make the points of different instance labels to repel each other in clustering to facilitate accurate instance segmentation. *L*_reg_ is the regularization loss which is used to make all cluster centers close to the origin of the feature space and help to form an effective and regular feature boundary for the embedding. The equations of *L*_*s*_, *L*_*d*_, *L*_reg_ are given as follows:

(6) $$\begin{array}{c} L_{s}=\frac{1}{I}\overset{I}{\underset{i=1}{\sum }}\frac{1}{N_{i}}\overset{N_{i}}{\underset{j=1}{\sum }}{\left[\max\left[0,{\left\|c_{i}-f_{j}\right\|}_{2}-\delta_{s}\right]\right]}^{2}, \end{array}$$

(7) $$\begin{array}{c} L_{d}=\frac{1}{\left(\begin{array}{c} I\\ 2 \end{array}\right)}\overset{I}{\underset{i_{A}=1}{\sum }}\overset{I}{\underset{\begin{array}{c} i_{B}=1\\ i_{B}\neq i_{A} \end{array}}{\sum }}{\left[\max\left[0,2\delta_{d}-{\left\|c_{i_{A}}-c_{i_{B}}\right\|}_{2}\right]\right]}^{2}, \end{array}$$

(8) $$\begin{array}{c} L_{\text{reg}}=\frac{1}{I}\overset{I}{\underset{i=1}{\sum }}{\left\|c_{i}\right\|}_{2}, \end{array}$$

where *I* represents the number of instances in the current point cloud batch being processed and *N*_*i*_ represents the number of points contained in the *i*-th instance; **c**_*i*_ represents the center of the points belonging to the *i*-th instance in the current feature space, and **f**_*j*_ represents the feature vector of the point *j* in the current feature space. The parameter *δ*_*s*_ defines a boundary threshold that allows the aggregation of points of the same instance, and 2*δ*_*d*_ represents the nearest feature distance threshold of two different instances.

In addition, in the output feature *F*_DGF_ of the DGFFM, in order to help integrating coarse-grained features and fine-grained features, it is also necessary to impose a supervision on this midlevel feature space. The purpose is to constrain the features belonging to the same instance to come closer in advance, while the features belonging to different instances to drift apart. Reference [69] proposed a point-level Double-hinge Loss (DHL) *L*_DHL_, which considered the constraints of the semantic task and the instance task on the middle-level features of the network. We directly transplanted DHL to the feature map *F*_DGF_; therefore, the total loss function of PSegNet can be represented as follows:

(9) $$\begin{array}{c} L=L_{\text{sem}}+L_{\text{ins}}+L_{\text{DHL}}. \end{array}$$

### 2.7. Evaluation Measures

In order to verify the semantic segmentation performance of PSegNet on the plant point cloud, we calculate four quantitative measures—Prec, Rec, F1, and Intersection over Union (IoU) for each semantic class, respectively. For all the four semantic measures (represented in percentage), the higher means the better. The measure Prec means precision, and it is the proportion of the points correctly classified in this semantic class to all the points predicted by the network. The notation Rec means recall, which reflects the proportion of the points correctly classified in this semantic class to the total points of this class in the ground truth. IoU reflects the degree of overlapping between the predicted areas of each semantic category and the corresponding real areas, and F1 is a comprehensive indicator calculated as the harmonic average of Prec and Rec. The equations of the four quantitative measures are given in the first half of Table 2, in which TP represents the number of true positive points of the current semantic category, and FP represents the false positive points of the current category, while FN stands for the false negative points.

The measures—mCov, mWCov, mPrec, and mRec, were used to evaluate the results of instance segmentation, and the equations of the four measures are defined in the second half of Table 2, respectively. In Table 2, IG_*m*_ represents the ground truth point set of the *m*-th instance under the same semantic class; IP_*n*_ represents the predicted point set of the *n*-th instance under the same semantic class. max[·] represents the maximum value of all terms evaluated. The binary function IoU[·, ·], which accepts two inputs as the point set of ground truth and the predicted point set from the network, is calculated exactly as the semantic IoU equation. The parameter *C* is the number of semantic classes for calculation of the Mean Precision (mPrec) and the Mean Recall (mRec). Because the dataset has three plant species, the semantic classes include the stem class and the leaf class of each plant species, which fixes *C* at 6. The notation |*TP*(sem = *i*)| represents the number of predicted instances whose IoU is above 0.5 in the semantic class *i*. The notation |IP(sem = *i*)| represents the total predicted number of instances in semantic class *i*. |IG(sem = *i*)| represents the number of instances of the ground truth in semantic class *i*.

## 3. Experiments and Results

### 3.1. Data Preparation and Training Details

**Figure 4:**
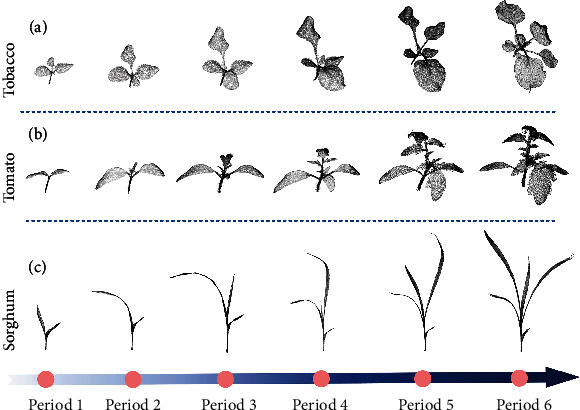
Demonstration of some point clouds from our dataset. (a) Point clouds in 6 consecutive growth periods of the same tobacco plant, respectively; (b) point clouds in 6 consecutive growth periods of the same tomato plant; (c) point clouds in consecutive 6 growth periods of the same sorghum plant.

The plant point cloud data used in this work originates from a laser-scanned point cloud dataset in [85, 86]. The dataset recorded three kinds of crops including tomato, tobacco, and sorghum growing in several different environments. Each crop was scanned multiple times during 30 days. We show the three crops of different growth periods in Figure 4. The scanning error of the dataset is controlled within ±25*μ*m. The dataset contains a total of 546 individual point clouds including 312 tomato point clouds, 105 tobacco point clouds, and 129 sorghum point clouds. The largest point cloud contains more than 100000 points, and the smallest has about 10000 points. We applied Semantic Segmentation Editor (SSE) [87] to annotate leaf and stem for semantic labels and the instance label for each single leaf. To be more specific, for semantic annotation, we classify the stem system and leaves of different species as different semantic categories. Therefore, in our dataset, there are six semantic categories with *C* = 6.

In order to prepare the data for network training and testing, the dataset should be divided and extended. Firstly, we divide the original dataset into the training set and test set under the ratio 2 : 1. The original dataset contains 546 point clouds, and for each point cloud, we used the VFPS method introduced in Section 2.1 to downsample it to a point cloud of *N* = 4096, and we repeated the downsampling for 10 times with randomly selected initial point in the last step of VFPS to augment the dataset. The randomness of data augmentation comes from the difference on the initial iteration point of FPS after voxelization in VFPS. Therefore, each point cloud in the dataset contains 4096 points after augmentation, and for the augmented 10 clouds generated from the same original point cloud, despite their similarity on appearance, the distributions of local points are quite different. Finally, we form the training set with 3640 point clouds and the test set with 1820 point clouds.

**Figure 5:**
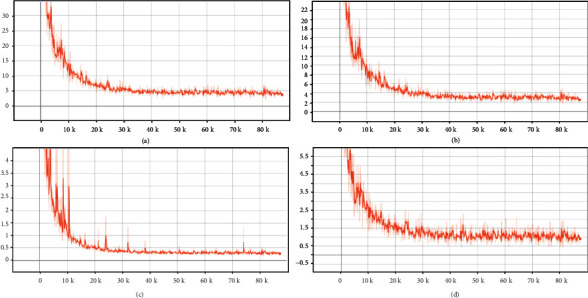
The changes of losses in the training of PSegNet. From (a–d) are the total loss *L*, the DHL *L*_DHL_ imposed on the midlevel feature layer after DGFMM, the semantic loss *L*_sem_, and the instance loss *L*_ins_ . The *x*-axis of all plots means the number of trained samples, and the *y*-axis is the loss value. Given 3640 training samples and the training batch size at 8, we have 455 samples to be trained in each epoch. When the training stops at 190 epochs, the *x*-axis ends at 455∗190 = 86450.

All the annotation work, training, testing, and the comparative experiments were conducted on a server under the Ubuntu 20.4 operating system. Our hardware platform contains an AMD RYZEN 2950x CPU that has 16 cores and 32 threads, a memory of 128 GB, and a GPU of NVIDIA RTX 2080Ti. The deep learning framework is TensorFlow 1.13.1. During training, batch size is fixed to 8, and the initial learning rate is set to 0.002; afterwards, the learning rate is reduced by 30% after 10 epochs per iteration. Adam solver is used to optimize our network, and Momentum is set to 0.9. For PSegNet and other methods compared, we end training at 190 epochs and record the model that has the minimum loss in testing as the selected model. In the encoder part of PSegNet, we set *k* = 16 in the first two DNFEBs and *k* = 8 for the last two DNFEBs for the KNN search range. The reasons of the KNN configuration are twofold. First, a large *K* brings a high calculation burden; therefore, *K* should not be a large number. Second, the number of features shrinks in the encoder part of PSegNet (from 4096 to 128) for encoding point cloud information efficiently. Thereby, the search range of the local KNN should also be declining with the shrinking features to keep the receptive field of the network stable. When building *L*_*s*_ and *L*_*d*_, we set *δ*_*s*_ = 0.5, and *δ*_*d*_ = 1.5. We also fixed *α* = *β* = 1 and *γ* = 0.001 throughout this study. We recoded the changes of losses of PSegNet during training; the values of the total loss and the three sublosses are displayed in Figure 5. All losses have shown an evident decline at first and a quick convergence.

### 3.2. Semantic Segmentation Results

**Figure 6:**
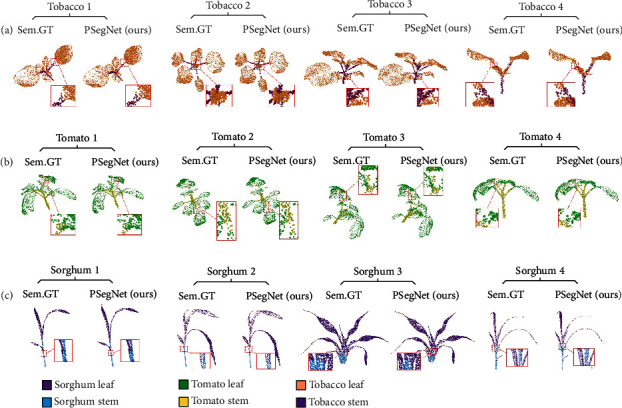
Qualitative demonstration of our PSegNet for semantic segmentation. (a) Semantic segmentation results of four different tobacco individuals, respectively. (b) Semantic segmentation results of four different tomato plants, respectively. (c) Semantic segmentation results of four different sorghum plants, respectively. Each segmented crop point cloud from PSegNet is compared with its corresponding ground truth (Sem.GT). The meanings of different rendered colors are shown at the bottom of the figure. Some of the areas are enlarged to give more details.

Figure 6 presents qualitative semantic results of PSegNet on three crop species. In order to reveal the real performance, we especially show test samples from different growth environments and stages. From Figure 6, we can see good segmentation results on all three crops. PSegNet seems to be good at determining the boundary between the stem and the leaves, because only rare false segmentations can be observed around the boundary between the two semantic classes.

Table 3 presents the quantitative semantic segmentation results of PSegNet for the total test set, on which most measures have reached above 85.0%, showing satisfactory semantic segmentation performance. From the measures listed in Table 3, it is not hard to observe that the leaf segmentation results of PSegNet are better than the stem results, and this is because the point number of stems is much fewer than leaves in the training data. Across the three species, tomato has the best semantic segmentation result, and the possible reason is that the tomato point clouds account for the largest proportion in the total training dataset. This imbalance training can be improved by adding more data from the two other species.

### 3.3. Instance Segmentation Results

**Figure 7:**
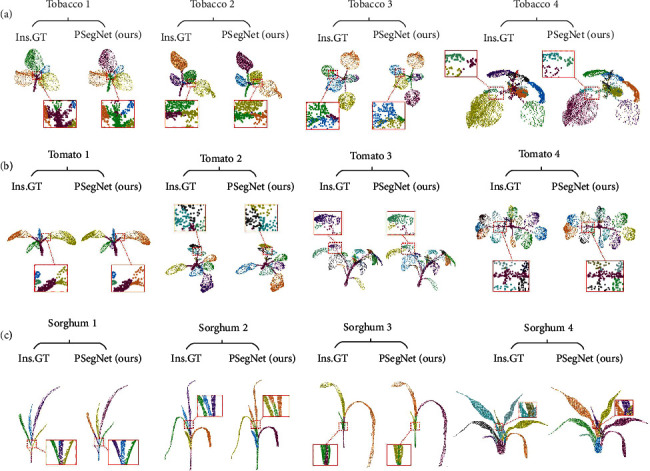
The qualitative demonstration of the instance segmentation by PSegNet. (a) Instance segmentation results of four different tobacco individuals, respectively. (b) Instance segmentation results of four different tomato individuals, respectively. (c) Instance segmentation results of four different sorghum individuals, respectively. Each segmented crop point cloud from PSegNet is compared with its corresponding ground truth (Ins.GT). Note that the different rendered colors in this figure are just for better visual separation of different instances, and it has no connection with the instance labels. Therefore, despite successful segmentation, the same leaf instance in the ground truth and the network result may be rendered with two different colors. Some of the areas are enlarged to give more details.

Figure 7 shows the qualitative evaluation of the instance results of three crops by PSegNet, and 12 representative point clouds from multiple growth stages are shown in Figure 7, respectively. Satisfactory segmentation of leaf instances can be observed on all three species in Figure 7. The three species differ heavily in leaf structure. Tobacco has big and broad leaves, and tomato plant has a compound leaf structure which contains at least one big leaflet and two small leaflets, while sorghum has long and slender leaves. PSegNet shows good leaf instance segmentation performance on all three types of leaves.

Table 4 lists the quantitative measures of leaf instance segmentation by PSegNet for the test set. Most of measures are above 80.0%, representing satisfactory instance segmentation performance.

### 3.4. Comparison with Other Methods

In this subsection, several mainstream point cloud segmentation networks are compared with our PSegNet on the same plant dataset. Among them, PointNet [64], PointNet++ [65], and DGCNN [74] are only capable of semantic segmentation. Like our network, ASIS [76] and PlantNet [77] can conduct the semantic segmentation and instance segmentation task simultaneously, and we use the same set of semantic and instance labels for training on the three dual-function networks. We used recommended parameter configurations for the comparative networks from their original papers, respectively.

Table 5 shows the quantitative comparison across six networks including PSegNet on the semantic segmentation task. PSegNet achieved the best in most cases and was superior to the others on all four averaged quantitative measures.  Table 6 shows the quantitative performance comparison of PSegNet with two dual-function segmentation networks (ASIS and PlantNet) on instance segmentation. Except for the mPrec, mRec, and mCov of sorghum leaves, our PSegNet has achieved the best performance at all cases including all the four averaged measures.

**Figure 8:**
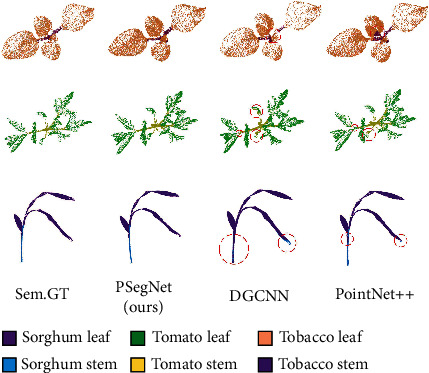
The qualitative semantic segmentation comparison on the three species. DGCNN and PointNet++ are compared with our PSegNet. The parts with segmentation errors are highlighted by red dotted circles, respectively. DGCNN and PointNet++ both have multiple prediction errors around the boundary between two different point classes.

**Figure 9:**
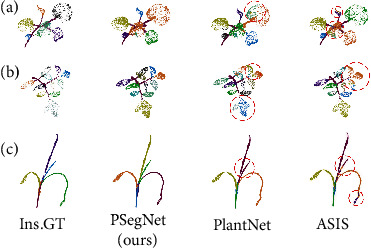
The qualitative instance segmentation comparison on the three species. (a) The tobacco plant and (b) the tomato plant; (c) the sorghum plant. PlantNet and ASIS are compared with our PSegNet. Note that the different rendered colors in this figure are just for better visual effect, and the colors are not associated with the instance labels. The parts with segmentation errors are highlighted by red dotted circles, respectively. PlantNet and ASIS both have multiple prediction errors around the boundaries of leaf instances.

We also compared PSegNet with state of the art qualitatively on both semantic segmentation and instance segmentation tasks. The qualitative semantic segmentation comparison on the three species is shown in Figure 8, and the instance segmentation comparison is shown in Figure 9. The samples in the two figures exhibit that PSegNet is superior to the networks that are specially designed for semantic segmentation, and PSegNet is also superior to the dual-function segmentation networks—ASIS and PlantNet for instance segmentation.

### 3.5. Ablation Study

In this section, we designed several independent ablation experiments to verify the effectiveness of the proposed modules in PSegNet, including VFPS, DNFEB, and DGFFM, as well as the SA and CA in the AM. The ablation experiments on semantic segmentation are shown in Table 7, and the ablation experiments on instance segmentation are shown in Table 8. In the two tables, the “Ver” column gives the version names of the ablated networks, respectively. Each version is formed by removing an existing module or some parts of a module from the original PSegNet. We compared seven versions of PSegNet named “A1” to “A7” with the complete PSegNet (named with “C”). In order to ensure the fairness of the experiments, ablating VFPS (A6) means using the basic FPS for downsampling and augmentation of the point cloud data. After ablating a module with convolutions, we will add MLPs with the same depth at the ablated position to ensure that the network depth (or the number of parameters) remains unchanged. For example, when ablating DNFEB, we replace it with a 6-layer MLP to form the A5 network. When ablating SA, we replace it with a 1-layer MLP to form the A2 network. When ablating DGFFM (A4), only the decoder branch that extracts fine-grained features is left to keep the depth of the network unchanged. In the A7 network, we only keep the stage 1 for all DNFEBs in PSegNet to validate the effectiveness of the 3-stage structure of DNFEB in feature extraction. In Table 7, the complete version of PSegNet has the best semantic segmentation performance in average, which proves the ablation of any proposed submodule will lead to the decline on the average segmentation performance and also indirectly verifies the effectiveness of all proposed submodules and the sampling strategy in the paper. In the ablation analysis experiments shown in Table 8, the complete PSegNet network has the best average instance segmentation performance. Ablating any proposed submodule will also lead to the decline of the average network segmentation performance, which again verifies the effectiveness of all proposed submodules.

## 4. Discussion

### 4.1. Generalization Ability of PSegNet

**Figure 10:**
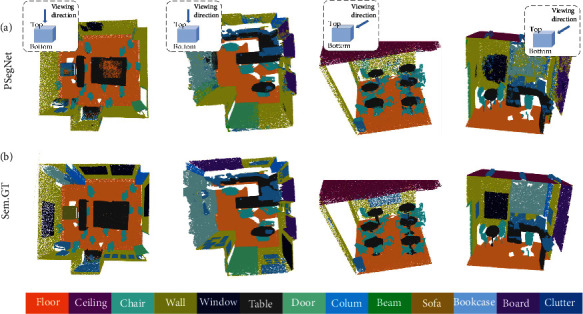
Demonstration of the semantic segmentation results of PSegNet on four different rooms in Area 5 of S3DIS. (a) The semantc prediction results from PSegNet and (b) the semantic ground truth. Different semantic classes are rendered with different colors at the bottom, respectively. The first two rooms are visualized from top, and the third and the fourth rooms are visualized with side views. Each room is composed of several 1 × 1 × he blocks.

**Figure 11:**
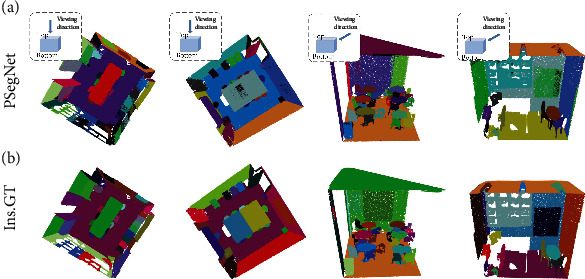
Demonstration of the instance segmentation results of PSegNet on four different rooms in Area 5 of S3DIS. (a) The instance prediction results from PSegNet and (b) the instance GT. Different instance classes are rendered with different colors, respectively. Note that the different rendered colors in this figure are just for better visual effect, and the colors are not associated with the instance labels. The first two rooms are visualized from top, and the third and the fourth rooms are visualized with side views. Each room is composed of several 1 × 1 × he blocks.

In this subsection, we prove that the proposed PSegNet is versatile enough to be applied to other types of point cloud data, not only restricted to 3D datasets for plant phenotyping. We trained and tested our PSegNet on the *Stanford Large-Scale 3D Indoor Spaces* (S3DIS) [88] to validate its applicability on point clouds of indoor scenes, which are very different from crops in 3D. The S3DIS dataset has 6 large indoor areas scanned by Lidars, including 271 rooms functioning as conference rooms, offices, and hallways. All points in the dataset are divided into 13 semantic classes such as floor, table, window, and so on. In addition, all points in each semantic class are labeled with instance indices. In training and testing, we cut each room into many 1 × 1 × he blocks measured in meter that do not overlap each other, and he means the height of each room. Each block was downsampled to 4096 points as a single point cloud input. The point clouds in Area 5 of S3DIS were used for testing, and the rest of the S3DIS areas were used for training. During training, we set all hyperparameters of PSegNet to be the same as the way the plant dataset was trained.  Figure 10 shows the qualitative semantic segmentation results of several S3DIS rooms, respectively. The majority of points were correctly classified by PSegNet comparing to the GT, and our network seems to be especially good at recognizing furniture such as tables and chairs with varied shapes and orientations. Instance segmentation on S3DIS is regarded as a challenging task; however, our PSegNet shows satisfactory instance segmentation on the four different rooms in Figure 11. PSegNet seems to have better instance segmentation on small objects than large objects; e.g., in the third room, PSegNet almost correctly labeled all single chairs.

### 4.2. Discussion of the Effectiveness

In this subsection, we would like to explain more about how this work handles the three challenges faced by current deep learning models for point cloud segmentation.

#### 4.2.1. Why the proposed VFPS strategy prevails?

**Figure 12:**
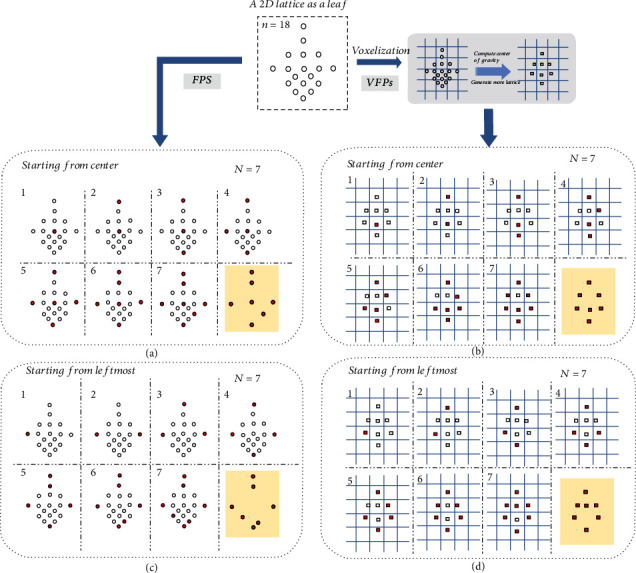
Comparison of VFPS and FPS on a 2D lattice like a leaf. The original lattice contains 18 points, sparse on the upper part and dense at the lower part. (a) The FPS (*N* = 7) process starting from the center; (b) the FPS (*N* = 7) process starting from the leftmost point; (c) VFPS (*N* = 7) process starting from the center point; (d) VFPS (*N* = 7) process starting from the leftmost point.

To better understand this, we constructed a simple 2D lattice with only 18 points to simulate a flat leaf in space and compared the difference between FPS and VFPS on the lattice. The 2D lattice, shown in Figure 12, was intentionally set to be sparse at the upper part while dense at the lower part. The aim of sampling for both FPS and VFPS is the same, to reduce the number of points to only 7. We fixed the number of voxels in VFPS as 8, which was slightly larger than the aim of downsampling (*N* = 7) according to the instruction of VFPS. Figure 12(a) shows the whole process of FPS starting from the center point of the lattice, and in the sampled 7 points, only one of them is from the interior part, and the other 6 are edge points. Figure 12(c) shows FPS starting from the leftmost point, and all 7 sampled points are located on the edge of lattice. Figures 12(a) and 12(c) reflect a common phenomenon of FPS that when *n* ≫ *N*, the sampling may concentrate on edges and easily create cavities on point clouds. And when *n* ≫ *N*, FPS also deviates from the common intuition that the low-density area gets points easier than the high-density area because the upper part of lattice of Figure 12(c) is not getting more points. Figures 12(b) and 12(d) show two different processes of VFPS on the voxelized lattice initialized with the center point and the leftmost point, respectively. The two downsampled VFPS results are smoother than the counterparts of FPS and have smaller cavities inside. In a real point cloud of crop, the leaves are usually flat and thin, presenting a similar structure as Figure 12 lattice in the 3D space. Moreover, we are frequently challenged with the sampling requirement of *n* ≫ *N* in 3D plant phenotyping, on which VPFS can generate smooth results with smaller cavities.

#### 4.2.2. How PSegNet strikes the balance between semantic segmentation and instance segmentation?

The network design for multifunctional point cloud segmentation is difficult. The reasons are twofold. First, each single segmentation task needs a specially designed network branch controlled by a unique loss function. Take PSegNet as the example, the semantic segmentation pathway and the instance segmentation pathway are restricted by *L*_sem_ and *L*_ins_, respectively. To better reconcile the training on the main network, we also added the point-level loss *L*_DHL_ to the feature map *F*_DGF_. Therefore, the total network of PSegNet is restricted by a combination of three losses. The second difficulty in design is that when adjusting the weight of a branch's loss in the total loss, the other branch will also be effected. For example, when increasing the proportion of the semantic loss in the PSegNet, the instance performance will likely drop. Thus, the balance between the semantic segmentation and the instance segmentation can be reached by controlling the assigned weights to their losses, respectively. Fortunately, after several tests, we found that the proportion of 1 : 1 for *L*_sem_ and *L*_ins_ was a choice good enough to outcompete state of the art.

#### 4.2.3. Why PSegNet has good generalization ability on plant species?

For a point cloud learning model, the bad generalization on species usually results in two undesirable phenomena. The first is that one may observe parts of plant species “A” on the point cloud of a plant species “B”; e.g., the model may falsely classify some points on the stem of a tomato plant as “the stem of a tobacco” in semantic segmentation. The second phenomenon is that one may see a big gap on segmentation performance between the monocotyledons (sorghum) and the dicotyledons (tomato and tobacco) due to the huge differences in 3D structure. In the qualitative and quantitative results of PSegNet, the two undesirable phenomena were rarely seen, and PSegNet outperformed popular networks on both semantic and instance segmentation tasks. In addition, we also have found that PSegNet has strong generalization ability on the object types in point clouds. The test of PSegNet on indoor S3DIS dataset (given in Section 4.1) proved that the network has potential to be generalized to other fields such as indoor SLAM (Simultaneous Localization and Mapping) and Self-Driving Vehicles.

The good generalization ability of PSegNet is most likely to come from the design of the network architecture in Figure 2. The DNFEB separately extracts high-level and low-level features from two local spaces and then aggregates them to realize better learning. DGFFM first uses two parallel decoders to construct feature layers with two different learning granularities, respectively. Then, DGFFM fuses the two feature granularities to create comprehensive feature learning. The AM part of PSegNet uses two types of attentions (spatial and channel) to lift the network training efficiency on both segmentation tasks.

### 4.3. Limitations

Although PSegNet can perform two kinds of segmentations well on three species with several growth periods, this network still does not work well on seedlings. For many plant species, the seedling period takes a very small and special 3D shape, which is different from all the other growth stages. Hence, the distinctiveness of the seedling period may cause problem in training and testing for deep learning networks.

The segmentation performance of PSegNet will decrease with the increasing complexity of the plant structure (e.g., trees), and unfortunately, this happens to all such networks. The reasons are twofold. First, the current dataset does not include plant samples with a lot of leaves; therefore, the network cannot work directly on samples of trees. Second, due to the restriction of hardware, the network only accepts 4096 points as one sample input. For a plant point cloud with dense foliage, the number of points on each organ will be very few, which causes sparse and bad feature learning and definitely outputs terrible segmentation results.

## 5. Conclusion

In this paper, we first proposed a Voxelized Farthest Point Sampling (VFPS) strategy. This new downsampling strategy for point clouds inherits merits from both the traditional FPS downsampling strategy and the VBS strategy. It can not only fix the number of points after downsampling but also able to augment the dataset with randomness, which renders it especially suitable for the training and testing of deep learning networks. Secondly, this paper designs a novel dual-function segmentation network—PSegNet; it is suitable for laser-scanned crop point clouds of multiple species. The end-to-end PSegNet is mainly composed of three parts—the front part has a typical encoder-like structure that is frequently observed in deep neural networks; the middle part is the Double-Granularity Feature Fusion Module (DGFFM), which decodes and integrates two features with different granularities. The third part of PSegNet has a double-flow structure with Attention Modules (AMs), in which the upper branch and the lower branch correspond to the instance segmentation task and semantic segmentation task, respectively. In qualitative and quantitative comparisons, our PSegNet outcompeted several popular networks including PointNet, PointNet++, ASIS, and PlantNet on both organ semantic and leaf instance segmentation tasks.

In the future, we will focus on two aspects. First, we will collect more crop point cloud data with high-precision and introduce more species (especially the monocotyledonous plants with slender organs) into the dataset as well. Secondly, we will devise new deep learning architectures that are more suitable for the understanding and processing of 3D plant structures and propose compressed networks with high segmentation accuracies to serve the real-time need in some scenarios of the agriculture industry.

## Figures and Tables

**Table 1:** Notations and nomenclatures.

FPS	Farthest Point Sampling
VBS	Voxelization-based Sampling
VFPS	Voxelized Farthest Point Sampling
DNFEB	Double-Neighborhood Feature Extraction Block
DGFFM	Double-Granularity Feature Fusion Module
AM	Attention Module
CA	Channel attention
SA	Spatial attention
DHL	Double-hinge Loss
GT	Ground truth
MLP	Multilayer perceptron
ReLU	Rectified linear unit activation
PE	Position encoding
EC	EdgeConv operation
AP	Attentive pooling

*F*_*c*_, *F*_*f*_	Feature maps after decoding
*F*_DGF_	Aggregated feature map after DGFFM
*L*, *L*_sem_, *L*_ins_, *L*_DHL_, *L*_*s*_, *L*_*d*_, *L*_reg_	The loss functions
*C*	The number of semantic classes
*N*	The number of points in a point cloud
**p**_*i*_	A point in *XYZ* space
**f**_*i*_, **r**_*i*_, **h**_*i*_, **f**_*i*_′, **fe**_*i*_	A point vector in feature space
*K*	The parameter of KNN
*α*, *β*, *γ*, *δ*_*s*_, *δ*_*d*_	Parameters for loss functions
∪	Feature concatenation
max[∙]	The maximum value across the inputs
IoU[∙, ∙]	IoU of the two entities
MLP[∙]	MLP operation with shared parameters

**Table 2:** The quantitative measures used in this work.

	Measures	Equations
Semantic segmentation	Prec	$\frac{\text{TP}}{\text{TP}+\text{FP}}$
	Rec	$\frac{\text{TP}}{\text{TP}+\text{FN}}$
	F1	$2\cdot \frac{\text{Prec}\cdot \text{Rec}}{\text{Prec}+\text{Rec}}$
	IoU	$\frac{\text{TP}}{\text{TP}+\text{FP}+\text{FN}}$

Instance segmentation	mCov	$\frac{1}{\text{I}}\overset{I}{\underset{m=1}{\sum }}\underset{n}{\max}\left[\text{IoU}\left[\text{I}{\text{G}}_{m},\text{I}{\text{P}}_{n}\right]\right]$
	mWCov	$\overset{I}{\underset{m=1}{\sum }}w_{m}\underset{n}{\max}\left[\text{IoU}\left[\text{I}{\text{G}}_{m},\text{I}{\text{P}}_{n}\right]\right]$
	mPrec	$\frac{1}{C}\overset{C}{\underset{i=1}{\sum }}\frac{\left|\text{TP}\left(\text{sem}=i\right)\right|}{\left|\text{IP}\left(\text{sem}=i\right)\right|}$
	mRec	$\frac{1}{C}\overset{C}{\underset{i=1}{\sum }}\frac{\left|\text{TP}\left(\text{sem}=i\right)\right|}{\left|\text{IG}\left(\text{sem}=i\right)\right|}$

**Table 3:** The quantitative measures of PSegNet on semantic segmentation.

	Tobacco		Tomato		Sorghum	
	Stem	Leaf	Stem	Leaf	Stem	Leaf
Prec (%)	92.71	96.76	96.36	97.98	89.54	98.04
Rec (%)	87.42	97.73	95.02	98.59	85.69	98.63
F1 (%)	89.99	97.24	95.68	98.29	87.57	98.33
IoU (%)	81.79	94.63	91.73	96.63	77.89	96.72

**Table 4:** The quantitative measures of PSegNet on instance segmentation.

	Tobacco leaf	Tomato leaf	Sorghum leaf
mPrec (%)	87.80	89.92	86.68
mRec (%)	77.09	78.62	82.13
mCov (%)	80.88	84.89	84.27
mWCov (%)	90.72	90.45	87.46

**Table 5:** Quantitative comparison of semantic segmentation performances of six networks including PSegNet.

	Methods	Tobacco		Tomato		Sorghum		Mean
		Stem	Leaf	Stem	Leaf	Stem	Leaf	
Prec (%)	PointNet	77.15	94.02	93.99	96.71	77.87	95.37	89.19
	PointNet++	87.78	95.62	93.65	96.80	78.01	**98.33**	91.70
	ASIS	91.65	91.94	93.55	97.14	85.47	95.17	92.49
	PlantNet	89.45	**96.80**	95.90	96.30	89.07	97.43	94.16
	DGCNN	90.55	96.42	95.24	97.86	83.95	97.37	93.57
	PSegNet (ours)	**92.71**	96.76	**96.36**	**97.98**	**89.54**	98.04	**95.23**

Rec (%)	PointNet	79.20	93.31	91.85	97.61	61.45	97.85	86.88
	PointNet++	**90.83**	94.05	92.45	97.33	78.66	98.27	91.93
	ASIS	83.85	96.11	92.87	95.51	81.65	97.88	91.31
	PlantNet	86.12	92.97	**95.24**	98.23	**86.06**	98.07	92.78
	DGCNN	85.55	**97.76**	94.15	98.27	78.05	98.20	92.00
	PSegNet (ours)	87.42	97.73	95.02	**98.59**	85.69	**98.63**	**93.85**

F1 (%)	PointNet	78.16	93.66	92.91	97.16	68.69	96.59	87.86
	PointNet++	89.28	94.83	93.05	97.07	78.33	98.30	91.81
	ASIS	87.58	93.98	93.21	96.32	83.52	96.51	91.85
	PlantNet	87.75	94.85	95.56	97.26	87.54	97.75	93.45
	DGCNN	87.98	97.09	94.69	98.07	80.89	97.78	92.75
	PSegNet (ours)	**89.99**	**97.24**	**95.68**	**98.29**	**87.57**	**98.33**	**94.52**

IoU (%)	PointNet	64.15	88.08	86.76	94.48	52.31	93.41	79.87
	PointNet++	80.63	90.17	87.00	94.30	64.38	96.65	85.52
	ASIS	77.91	88.64	87.29	92.90	71.70	93.25	85.28
	PlantNet	78.17	90.20	91.51	94.66	77.84	95.59	88.00
	DGCNN	78.54	94.34	89.92	96.20	76.92	95.65	88.60
	PSegNet (ours)	**81.79**	**94.63**	**91.73**	**96.63**	**77.89**	**96.72**	**89.90**

The best values are in boldface.

**Table 6:** Quantitative comparison of instance segmentation performances of six networks including PSegNet.

	Methods	Tobacco leaf	Tomato leaf	Sorghum leaf	Mean
mPrec (%)	ASIS	78.54	80.21	79.04	79.26
	PlantNet	87.74	85.50	79.39	84.21
	PSegNet (ours)	**87.80**	**89.92**	**86.68**	**88.13**

mRec (%)	ASIS	56.27	64.84	72.08	64.40
	PlantNet	69.36	76.63	81.83	75.94
	PSegNet (ours)	**77.09**	**78.62**	**82.13**	**79.28**

mCov (%)	ASIS	62.88	76.61	74.26	71.25
	PlantNet	71.98	83.34	82.63	79.32
	PSegNet (ours)	**80.88**	**84.89**	**84.27**	**83.35**

mWCov (%)	ASIS	73.95	82.73	77.31	78.00
	PlantNet	84.83	89.48	85.68	86.66
	PSegNet (ours)	**90.72**	**90.45**	**87.46**	**89.54**

The best values are in boldface.

**Table 7:** The ablation analysis of PSegNet on semantic segmentation. The check mark stands for the use of a module. The best quantitative values are shown in bold. The sign “**○**” means the partial ablation of DNFEB.

	Ver	VFPS	DNFEB	DGFFM	MA		Tobacco		Tomato		Sorghum		Mean
					SA	CA	Stem	Leaf	Stem	Leaf	Stem	Leaf	
Prec (%)	A1	√	√	√	√		92.47	97.09	95.82	96.78	86.38	97.56	94.35
	A2	√	√	√		√	91.01	95.68	96.12	97.80	87.63	96.89	94.19
	A3	√	√	√			91.39	96.65	95.90	96.89	**90.31**	96.77	94.65
	A4	√	√		√	√	89.48	**97.29**	95.95	97.54	86.63	95.79	93.78
	A5	√		√	√	√	59.58	76.59	84.63	90.35	62.95	84.87	76.50
	A6		√	√	√	√	89.46	97.12	96.17	95.82	85.82	96.49	93.48
	A7	√	○	√	√	√	86.43	97.07	94.65	97.47	88.44	97.18	93.54
	C	√	√	√	√	√	**92.71**	96.76	**96.36**	**97.98**	89.54	**98.04**	**95.23**

Rec (%)	A1	√	√	√	√		**88.00**	94.46	**95.41**	98.24	**87.59**	97.58	93.55
	A2	√	√	√		√	86.68	96.24	94.79	98.04	84.26	98.28	93.05
	A3	√	√	√			87.54	92.69	95.20	98.11	87.36	**98.90**	93.30
	A4	√	√		√	√	85.88	94.23	94.49	**98.60**	85.22	97.90	92.72
	A5	√		√	√	√	59.18	77.13	81.02	90.59	52.10	88.49	74.75
	A6		√	√	√	√	87.49	90.02	94.95	98.59	84.56	97.69	92.22
	A7	√	○	√	√	√	82.86	95.26	94.21	98.47	85.48	97.98	92.38
	C	√	√	√	√	√	87.42	**97.73**	95.02	98.59	85.69	98.63	**93.85**

F1 (%)	A1	√	√	√	√		**90.18**	95.75	95.61	97.51	86.98	97.57	93.93
	A2	√	√	√		√	88.79	95.96	95.45	97.92	85.91	97.58	93.60
	A3	√	√	√			89.42	94.63	95.55	97.50	**88.81**	97.82	93.96
	A4	√	√		√	√	87.64	95.74	95.22	98.07	85.92	96.84	93.24
	A5	√		√	√	√	59.38	76.86	82.79	90.47	57.01	86.64	75.53
	A6		√	√	√	√	88.46	93.44	95.55	97.18	85.19	97.09	92.82
	A7	√	○	√	√	√	84.61	96.16	94.43	97.97	86.94	97.58	92.95
	C	√	√	√	√	√	89.99	**97.24**	**95.68**	**98.29**	87.57	**98.33**	**94.52**

IoU (%)	A1	√	√	√	√		**82.11**	91.85	91.60	95.13	76.96	95.25	88.82
	A2	√	√	√		√	79.84	92.23	91.30	95.92	75.30	95.27	88.31
	A3	√	√	√			80.87	89.81	91.47	95.12	**79.87**	95.74	88.81
	A4	√	√		√	√	78.01	91.82	90.87	96.21	75.32	93.87	87.68
	A5	√		√	√	√	42.22	62.42	70.63	82.60	39.87	76.43	62.36
	A6		√	√	√	√	79.31	87.68	91.48	94.52	74.20	94.34	86.92
	A7	√	○	√	√	√	73.32	92.60	89.44	96.02	76.89	95.27	87.26
	C	√	√	√	√	√	81.79	**94.63**	**91.73**	**96.63**	77.89	**96.72**	**89.90**

**Table 8:** The ablation analysis of PSegNet on instance segmentation. The check mark stands for the use of a module. The best quantitative values are shown in bold. The sign “**○**” means the partial ablation of DNFEB.

	Ver	VFPS	DNFEB	DGFFM	MA		Tobacco leaf	Tomato leaf	Sorghum leaf	Mean
					SA	CA				
mPrec (%)	A1	√	√	√	√		86.98	88.60	77.53	84.37
	A2	√	√	√		√	86.44	90.50	82.28	86.41
	A3	√	√	√			90.00	88.07	79.98	86.02
	A4	√	√		√	√	87.93	89.02	77.78	84.91
	A5	√		√	√	√	64.19	76.23	38.73	59.72
	A6		√	√	√	√	**89.54**	89.08	79.72	86.11
	A7	√	○	√	√	√	87.83	88.19	83.64	86.55
	C	√	√	√	√	√	87.80	**89.92**	**86.68**	**88.13**

mRec (%)	A1	√	√	√	√		73.65	79.23	81.29	78.06
	A2	√	√	√		√	74.57	77.44	79.31	77.11
	A3	√	√	√			73.09	**79.41**	80.30	77.60
	A4	√	√		√	√	73.12	77.72	77.13	75.99
	A5	√		√	√	√	43.16	41.18	36.73	40.36
	A6		√	√	√	√	70.50	74.88	78.42	74.60
	A7	√	○	√	√	√	75.46	77.83	80.00	77.76
	C	√	√	√	√	√	**77.09**	78.62	**82.13**	**79.28**

mCov (%)	A1	√	√	√	√		76.73	85.06	82.49	81.43
	A2	√	√	√		√	78.87	84.34	81.80	81.67
	A3	√	√	√			75.38	**85.34**	83.11	81.28
	A4	√	√		√	√	77.27	84.20	81.75	81.07
	A5	√		√	√	√	44.50	58.10	42.09	48.23
	A6		√	√	√	√	74.83	82.51	81.61	79.65
	A7	√	○	√	√	√	77.51	84.57	81.21	81.10
	C	√	√	√	√	√	**80.88**	84.89	**84.27**	**83.35**

mWCov (%)	A1	√	√	√	√		87.72	90.24	86.31	88.09
	A2	√	√	√		√	89.77	89.78	86.00	88.52
	A3	√	√	√			86.85	90.06	86.48	87.80
	A4	√	√		√	√	87.92	89.60	85.32	87.61
	A5	√		√	√	√	64.04	71.86	49.17	61.69
	A6		√	√	√	√	84.29	88.64	85.67	86.20
	A7	√	○	√	√	√	87.67	89.52	84.47	87.22
	C	√	√	√	√	√	**90.72**	**90.45**	**87.46**	**89.54**

## Data Availability

The data and the code are available upon request.
